# *N*-[3-Chloro-4-(3-fluoro­benz­yloxy)phen­yl]-6-iodo­quinazolin-4-amine

**DOI:** 10.1107/S1600536810021847

**Published:** 2010-06-26

**Authors:** Zhi-Qiang Cai, Jing-Guo Liu, Wei-Wei Zhou, Yi-Liang Li

**Affiliations:** a Tianjin Key Laboratory of Drug Design and Discovery, Tianjin Institute of Pharmaceutical Research, Tianjin 300193, People’s Republic of China; b Faculty of Pharmacy, GuangXi Traditional Chinese Medical University, Nanning 530001, People’s Republic of China

## Abstract

In the title mol­ecule, C_21_H_14_ClFIN_3_O, the bicyclic ring system has a twisted conformation; the two fused rings form a dihedral angle of 4.5 (1)°. The dihedral angles between the fused ring system and the benzene rings are 27.3 (6) and 5.3 (5)° while the dihedral angle between the benzene rings is 22.0 (5)°. In the crystal structure, weak inter­molecular N—H⋯N hydrogen bonds link the mol­ecules into chains propagating in [100]. A short inter­molecular distance of 3.806 (3) Å between the centroids of the fluorobenzene and iodobenzene rings suggests the existence of π–π stacking inter­actions.

## Related literature

For a related structure, see: Calestani *et al.* (2001 ▶). The title compound is an important inter­mediate in the synthesis of the anti­cancer agent lapatinib, see: Kimberly *et al.* (2006 ▶).
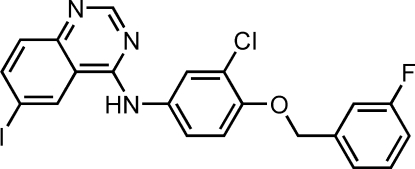


## Experimental

### 

#### Crystal data

C_21_H_14_ClFIN_3_O
*M* *_r_* = 505.70
Orthorhombic, 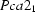

*a* = 13.128 (3) Å
*b* = 7.6293 (15) Å
*c* = 18.898 (4) Å
*V* = 1892.8 (7) Å^3^
*Z* = 4
Mo *K*α radiation
μ = 1.86 mm^−1^
*T* = 113 K
0.20 × 0.18 × 0.06 mm

#### Data collection

Rigaku Saturn CCD area-detector diffractometer
Absorption correction: multi-scan (*ABSCOR*; Higashi, 1995 ▶) *T*_min_ = 0.707, *T*_max_ = 0.897
11905 measured reflections
3183 independent reflections
2510 reflections with *I* > 2σ(*I*)
*R*_int_ = 0.045

#### Refinement

*R*[*F* ^2^ > 2σ(*F* ^2^)] = 0.030
*wR*(*F* ^2^) = 0.067
*S* = 1.01
3183 reflections
258 parameters
1 restraint
H atoms treated by a mixture of independent and constrained refinement
Δρ_max_ = 1.24 e Å^−3^
Δρ_min_ = −0.70 e Å^−3^
Absolute structure: Flack (1983 ▶), 1433 Friedel pairs
Flack parameter: −0.039 (19)

### 

Data collection: *RAPID-AUTO* (Rigaku, 1998 ▶); cell refinement: *RAPID-AUTO*; data reduction: *CrystalStructure* (Rigaku/MSC, 2002 ▶); program(s) used to solve structure: *SHELXS97* (Sheldrick, 2008 ▶); program(s) used to refine structure: *SHELXL97* (Sheldrick, 2008 ▶); molecular graphics: *SHELXTL* (Sheldrick, 2008 ▶); software used to prepare material for publication: *SHELXTL*.

## Supplementary Material

Crystal structure: contains datablocks I, global. DOI: 10.1107/S1600536810021847/cv2724sup1.cif

Structure factors: contains datablocks I. DOI: 10.1107/S1600536810021847/cv2724Isup2.hkl

Additional supplementary materials:  crystallographic information; 3D view; checkCIF report

## Figures and Tables

**Table 1:** Hydrogen-bond geometry (Å, °)

*D*—H⋯*A*	*D*—H	H⋯*A*	*D*⋯*A*	*D*—H⋯*A*
N1—H21⋯N3^i^	0.81 (6)	2.39 (6)	3.128 (6)	151 (6)

Symmetry code: (i) 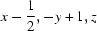.

**Fig. 1.**
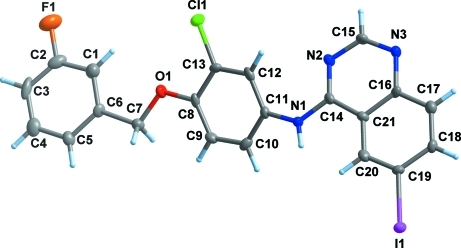
The structure of C21H14ClFIN3O with atom-labelling scheme and ellipsoids drawn at the 50% probability level.

## supplementary crystallographic information

### Comment

The title compound (I) is an important intermediate in the preparation of anticancer agent lapatinib (Kimberly *et al.*, 2006). Herein, the synthesis and the crystal structure of (I) are reported.

In (I) (Fig. 1), all bond lengths and angles are normal and comparable with those observed in the related compound (Calestani *et al.*, 2001). The bicycle quinazoline system has a twisted conformation - two fused rings form a dihedral angle of 4.5 (1)°. In the crystal structure, weak intermolecular N—H···N hydrogen bonds (Table 1) link molecules into chains propagated in direction [100]. Short intermolecular distance of 3.806 (3) Å between the centroids of aromatic rings suggests an existence of π-π interactions.

### Experimental

2-Chloro-4-(6-iodo-quinazolin-4-ylamino)-phenol (10 mmol) in acetone (5 ml) was added to a stirred mixture of anhydrous potassium carbonate (20 mmol) and 1-Chloromethyl-3-fluoro-benzene (10 mmol) in dry acetone (25 ml). It was stirred at room temperature for 6 h. Upon completion reaction mixture was diluted with water, extracted with dichloromethane and concentrated. Recrystallization from ethyl acetate afforded title compound in 89% yield as yellow crystal: 1H NMR (DMSO-d6): 9.82 (1*H*, s, NH), 8.94(1*H*, s, ArH), 8.60(1*H*, s, ArH), 8.08(1*H*, dd, ArH), 8.01(1*H*, d ArH), 7.72 (1*H*, dd ArH), 7.49(1*H*, d ArH), 7.43 (1*H*, dd ArH), 7.19 (3*H*, m ArH), 7.14 (1*H*, t ArH), 5.24(2*H*, s CH2).

### Refinement

All H atoms were initially located in a difference Fourier map. C-bound H atoms were then constrained to an ideal geometry (C—H  0.93 Å), N-bound H atom was refined with N—H bond restraint of 0.83 (5) Å. All H-atoms were refined with *U*_iso_(H) = 1.2*U*_eq_(C,*N*).

### Crystal data

**Table:**

C_21_H_14_ClFIN_3_O	*F*(000) = 992
*M**_r_* = 505.70	*D*_x_ = 1.775 Mg m^−^^3^
Orthorhombic, *P**c**a*2_1_	Mo *K*α radiation, λ = 0.71073 Å
Hall symbol: P 2c -2ac	Cell parameters from 7022 reflections
*a* = 13.128 (3) Å	θ = 1.1–27.9°
*b* = 7.6293 (15) Å	µ = 1.86 mm^−^^1^
*c* = 18.898 (4) Å	*T* = 113 K
*V* = 1892.8 (7) Å^3^	Prism, colourless
*Z* = 4	0.20 × 0.18 × 0.06 mm

### Data collection

**Table:**

Rigaku Saturn CCD area-detector diffractometer	3183 independent reflections
Radiation source: fine-focus sealed tube	2510 reflections with *I* > 2σ(*I*)
graphite	*R*_int_ = 0.045
Detector resolution: 14.63 pixels mm^-1^	θ_max_ = 25.0°, θ_min_ = 3.1°
ω scan	*h* = −15→15
Absorption correction: multi-scan (*ABSCOR*; Higashi, 1995)	*k* = −8→9
*T*_min_ = 0.707, *T*_max_ = 0.897	*l* = −21→22
11905 measured reflections	

### Refinement

**Table:**

Refinement on *F*^2^	Hydrogen site location: inferred from neighbouring sites
Least-squares matrix: full	H atoms treated by a mixture of independent and constrained refinement
*R*[*F*^2^ > 2σ(*F*^2^)] = 0.030	*w* = 1/[σ^2^(*F*_o_^2^) + (0.0292*P*)^2^] where *P* = (*F*_o_^2^ + 2*F*_c_^2^)/3
*wR*(*F*^2^) = 0.067	(Δ/σ)_max_ = 0.001
*S* = 1.00	Δρ_max_ = 1.24 e Å^−^^3^
3183 reflections	Δρ_min_ = −0.70 e Å^−^^3^
258 parameters	Extinction correction: *SHELXL97* (Sheldrick, 2008), Fc^*^=kFc[1+0.001xFc^2^λ^3^/sin(2θ)]^-1/4^
1 restraint	Extinction coefficient: 0.0309 (8)
Primary atom site location: structure-invariant direct methods	Absolute structure: Flack (1983), 1433 Friedel pairs
Secondary atom site location: difference Fourier map	Flack parameter: −0.039 (19)

### Special details

**Table:**

Geometry. All e.s.d.'s (except the e.s.d. in the dihedral angle between two l.s. planes) are estimated using the full covariance matrix. The cell e.s.d.'s are taken into account individually in the estimation of e.s.d.'s in distances, angles and torsion angles; correlations between e.s.d.'s in cell parameters are only used when they are defined by crystal symmetry. An approximate (isotropic) treatment of cell e.s.d.'s is used for estimating e.s.d.'s involving l.s. planes.
Refinement. Refinement of *F*^2^ against ALL reflections. The weighted *R*-factor *wR* and goodness of fit *S* are based on *F*^2^, conventional *R*-factors *R* are based on *F*, with *F* set to zero for negative *F*^2^. The threshold expression of *F*^2^ > σ(*F*^2^) is used only for calculating *R*-factors(gt) *etc*. and is not relevant to the choice of reflections for refinement. *R*-factors based on *F*^2^ are statistically about twice as large as those based on *F*, and *R*- factors based on ALL data will be even larger.

### Fractional atomic coordinates and isotropic or equivalent isotropic displacement parameters (Å^2^)

**Table:**

	*x*	*y*	*z*	*U*_iso_*/*U*_eq_	
I1	0.299873 (18)	−0.00631 (3)	0.50844 (4)	0.01983 (11)	
Cl1	0.28694 (8)	1.26802 (14)	0.25075 (8)	0.0248 (3)	
F1	0.0085 (3)	1.7039 (4)	0.0746 (2)	0.0538 (10)	
O1	0.0946 (2)	1.1497 (4)	0.19493 (17)	0.0220 (8)	
N1	0.2954 (3)	0.6395 (5)	0.3538 (2)	0.0165 (9)	
N2	0.4616 (3)	0.7425 (5)	0.3551 (2)	0.0185 (10)	
N3	0.6006 (3)	0.5682 (5)	0.3991 (2)	0.0173 (9)	
C1	0.0028 (5)	1.4220 (8)	0.1242 (3)	0.0289 (14)	
H1	0.0727	1.4259	0.1319	0.035*	
C2	−0.0476 (4)	1.5583 (9)	0.0935 (3)	0.0288 (16)	
C3	−0.1501 (5)	1.5661 (7)	0.0807 (3)	0.0336 (14)	
H3	−0.1804	1.6640	0.0603	0.040*	
C4	−0.2058 (4)	1.4207 (7)	0.0999 (3)	0.0290 (13)	
H4	−0.2756	1.4188	0.0914	0.035*	
C5	−0.1602 (3)	1.2779 (6)	0.1315 (3)	0.0225 (12)	
H5	−0.1995	1.1821	0.1449	0.027*	
C6	−0.0549 (4)	1.2766 (6)	0.1433 (3)	0.0171 (12)	
C7	−0.0070 (3)	1.1135 (6)	0.1729 (3)	0.0201 (11)	
H7A	−0.0466	1.0720	0.2129	0.024*	
H7B	−0.0066	1.0223	0.1371	0.024*	
C8	0.1419 (4)	1.0178 (5)	0.2308 (3)	0.0177 (11)	
C9	0.1065 (4)	0.8496 (6)	0.2378 (3)	0.0193 (11)	
H9	0.0455	0.8177	0.2163	0.023*	
C10	0.1605 (4)	0.7262 (6)	0.2767 (2)	0.0210 (12)	
H10	0.1359	0.6121	0.2797	0.025*	
C11	0.2503 (3)	0.7694 (6)	0.3110 (2)	0.0138 (10)	
C12	0.2883 (4)	0.9387 (7)	0.3038 (3)	0.0175 (11)	
H12	0.3480	0.9709	0.3268	0.021*	
C13	0.2365 (4)	1.0605 (5)	0.2618 (3)	0.0168 (11)	
C14	0.3949 (3)	0.6194 (6)	0.3725 (2)	0.0158 (10)	
C15	0.5600 (4)	0.7107 (7)	0.3715 (3)	0.0202 (13)	
H15	0.6052	0.8015	0.3620	0.024*	
C16	0.5303 (4)	0.4390 (8)	0.4183 (3)	0.0164 (13)	
C17	0.5680 (4)	0.2842 (6)	0.4475 (3)	0.0209 (12)	
H17	0.6380	0.2679	0.4516	0.025*	
C18	0.5028 (3)	0.1559 (6)	0.4703 (2)	0.0179 (10)	
H18	0.5282	0.0517	0.4887	0.022*	
C19	0.3977 (3)	0.1828 (5)	0.4656 (2)	0.0156 (10)	
C20	0.3593 (3)	0.3317 (5)	0.4348 (3)	0.0170 (11)	
H20	0.2893	0.3468	0.4307	0.020*	
C21	0.4254 (4)	0.4606 (6)	0.4094 (3)	0.0158 (11)	
H21	0.260 (5)	0.555 (7)	0.363 (4)	0.05 (2)*	

### Atomic displacement parameters (Å^2^)

**Table:**

	*U*^11^	*U*^22^	*U*^33^	*U*^12^	*U*^13^	*U*^23^
I1	0.01930 (17)	0.01590 (15)	0.02429 (18)	−0.00277 (13)	0.0006 (2)	0.00282 (15)
Cl1	0.0276 (6)	0.0170 (6)	0.0299 (8)	−0.0025 (5)	−0.0030 (7)	0.0048 (5)
F1	0.065 (2)	0.034 (2)	0.062 (3)	−0.0141 (18)	−0.011 (2)	0.0118 (17)
O1	0.0168 (18)	0.0207 (18)	0.029 (2)	0.0031 (15)	−0.0055 (17)	0.0074 (15)
N1	0.013 (2)	0.014 (2)	0.022 (3)	−0.0002 (18)	0.0021 (18)	0.0039 (17)
N2	0.013 (2)	0.021 (2)	0.021 (3)	0.0003 (17)	0.0004 (17)	0.0033 (18)
N3	0.014 (2)	0.017 (2)	0.021 (3)	0.002 (2)	0.002 (2)	0.0030 (18)
C1	0.028 (3)	0.025 (3)	0.034 (4)	0.002 (3)	0.000 (3)	0.001 (3)
C2	0.046 (5)	0.022 (3)	0.019 (4)	−0.009 (3)	−0.007 (3)	0.001 (3)
C3	0.052 (4)	0.027 (3)	0.022 (3)	0.024 (3)	−0.013 (3)	−0.006 (2)
C4	0.035 (3)	0.033 (3)	0.019 (3)	0.010 (3)	−0.004 (2)	−0.005 (2)
C5	0.021 (3)	0.027 (3)	0.018 (3)	0.004 (2)	−0.005 (2)	0.001 (2)
C6	0.024 (3)	0.017 (3)	0.010 (3)	0.003 (2)	−0.003 (2)	0.004 (2)
C7	0.018 (3)	0.021 (3)	0.022 (3)	−0.001 (2)	−0.001 (2)	0.003 (2)
C8	0.015 (3)	0.022 (3)	0.016 (3)	0.008 (2)	0.002 (2)	0.005 (2)
C9	0.015 (2)	0.022 (3)	0.021 (3)	−0.003 (2)	−0.003 (2)	−0.002 (2)
C10	0.030 (3)	0.013 (2)	0.021 (3)	0.001 (2)	0.002 (2)	0.0024 (19)
C11	0.014 (2)	0.016 (2)	0.012 (3)	0.004 (2)	0.000 (2)	0.0000 (18)
C12	0.012 (3)	0.028 (3)	0.013 (3)	0.005 (2)	0.001 (2)	−0.002 (2)
C13	0.017 (3)	0.0134 (19)	0.020 (3)	−0.004 (2)	0.004 (2)	0.001 (3)
C14	0.013 (2)	0.017 (2)	0.017 (3)	0.002 (2)	0.001 (2)	−0.0035 (19)
C15	0.015 (3)	0.019 (3)	0.027 (4)	−0.003 (2)	0.005 (2)	0.004 (2)
C16	0.018 (3)	0.012 (3)	0.019 (4)	0.002 (2)	0.007 (2)	0.002 (2)
C17	0.019 (3)	0.021 (3)	0.022 (3)	0.003 (2)	0.000 (2)	−0.001 (2)
C18	0.020 (3)	0.015 (2)	0.019 (3)	0.005 (2)	−0.001 (2)	0.003 (2)
C19	0.017 (3)	0.014 (2)	0.016 (3)	−0.004 (2)	0.001 (2)	0.0003 (19)
C20	0.015 (3)	0.022 (3)	0.015 (3)	0.000 (2)	−0.003 (2)	−0.003 (2)
C21	0.013 (3)	0.019 (3)	0.016 (3)	−0.003 (2)	−0.001 (2)	0.0007 (19)

### Geometric parameters (Å, °)

**Table:**

I1—C19	2.094 (4)	C7—H7A	0.9700
Cl1—C13	1.729 (4)	C7—H7B	0.9700
F1—C2	1.380 (7)	C8—C9	1.372 (6)
O1—C8	1.363 (5)	C8—C13	1.411 (8)
O1—C7	1.425 (5)	C9—C10	1.389 (6)
N1—C14	1.362 (5)	C9—H9	0.9300
N1—C11	1.408 (6)	C10—C11	1.386 (6)
N1—H21	0.81 (6)	C10—H10	0.9300
N2—C14	1.326 (6)	C11—C12	1.392 (7)
N2—C15	1.350 (6)	C12—C13	1.398 (8)
N3—C15	1.318 (6)	C12—H12	0.9300
N3—C16	1.399 (7)	C14—C21	1.454 (7)
C1—C2	1.363 (9)	C15—H15	0.9300
C1—C6	1.391 (7)	C16—C17	1.395 (7)
C1—H1	0.9300	C16—C21	1.397 (7)
C2—C3	1.368 (8)	C17—C18	1.370 (6)
C3—C4	1.377 (9)	C17—H17	0.9300
C3—H3	0.9300	C18—C19	1.398 (6)
C4—C5	1.380 (7)	C18—H18	0.9300
C4—H4	0.9300	C19—C20	1.373 (6)
C5—C6	1.400 (6)	C20—C21	1.396 (7)
C5—H5	0.9300	C20—H20	0.9300
C6—C7	1.502 (6)		
			
C8—O1—C7	115.4 (4)	C11—C10—C9	121.4 (4)
C14—N1—C11	129.1 (4)	C11—C10—H10	119.3
C14—N1—H21	114 (5)	C9—C10—H10	119.3
C11—N1—H21	116 (5)	C10—C11—C12	118.6 (4)
C14—N2—C15	116.6 (4)	C10—C11—N1	117.3 (4)
C15—N3—C16	114.6 (4)	C12—C11—N1	124.0 (4)
C2—C1—C6	117.0 (6)	C11—C12—C13	119.9 (5)
C2—C1—H1	121.5	C11—C12—H12	120.0
C6—C1—H1	121.5	C13—C12—H12	120.0
C1—C2—C3	125.9 (6)	C12—C13—C8	120.7 (4)
C1—C2—F1	117.7 (5)	C12—C13—Cl1	119.4 (4)
C3—C2—F1	116.4 (6)	C8—C13—Cl1	119.9 (4)
C2—C3—C4	116.2 (6)	N2—C14—N1	119.3 (4)
C2—C3—H3	121.9	N2—C14—C21	121.8 (4)
C4—C3—H3	121.9	N1—C14—C21	118.9 (4)
C3—C4—C5	121.3 (5)	N3—C15—N2	128.8 (5)
C3—C4—H4	119.4	N3—C15—H15	115.6
C5—C4—H4	119.4	N2—C15—H15	115.6
C4—C5—C6	120.2 (5)	C17—C16—C21	119.8 (5)
C4—C5—H5	119.9	C17—C16—N3	117.7 (5)
C6—C5—H5	119.9	C21—C16—N3	122.4 (5)
C1—C6—C5	119.4 (5)	C18—C17—C16	120.5 (5)
C1—C6—C7	122.0 (5)	C18—C17—H17	119.8
C5—C6—C7	118.5 (4)	C16—C17—H17	119.8
O1—C7—C6	109.9 (4)	C17—C18—C19	119.5 (4)
O1—C7—H7A	109.7	C17—C18—H18	120.2
C6—C7—H7A	109.7	C19—C18—H18	120.2
O1—C7—H7B	109.7	C20—C19—C18	120.7 (4)
C6—C7—H7B	109.7	C20—C19—I1	120.6 (3)
H7A—C7—H7B	108.2	C18—C19—I1	118.7 (3)
O1—C8—C9	125.8 (5)	C19—C20—C21	120.1 (4)
O1—C8—C13	115.9 (4)	C19—C20—H20	120.0
C9—C8—C13	118.3 (4)	C21—C20—H20	120.0
C8—C9—C10	120.8 (5)	C20—C21—C16	119.2 (5)
C8—C9—H9	119.6	C20—C21—C14	125.4 (4)
C10—C9—H9	119.6	C16—C21—C14	115.3 (5)
			
C6—C1—C2—C3	1.0 (10)	O1—C8—C13—Cl1	−1.9 (7)
C6—C1—C2—F1	179.6 (5)	C9—C8—C13—Cl1	177.0 (4)
C1—C2—C3—C4	−1.1 (10)	C15—N2—C14—N1	−176.3 (5)
F1—C2—C3—C4	−179.8 (5)	C15—N2—C14—C21	1.9 (7)
C2—C3—C4—C5	1.3 (8)	C11—N1—C14—N2	6.6 (7)
C3—C4—C5—C6	−1.4 (8)	C11—N1—C14—C21	−171.6 (5)
C2—C1—C6—C5	−1.0 (8)	C16—N3—C15—N2	−5.0 (8)
C2—C1—C6—C7	175.8 (5)	C14—N2—C15—N3	4.3 (8)
C4—C5—C6—C1	1.2 (8)	C15—N3—C16—C17	179.7 (5)
C4—C5—C6—C7	−175.6 (5)	C15—N3—C16—C21	−0.6 (8)
C8—O1—C7—C6	172.2 (4)	C21—C16—C17—C18	−2.7 (8)
C1—C6—C7—O1	15.5 (7)	N3—C16—C17—C18	177.1 (5)
C5—C6—C7—O1	−167.8 (4)	C16—C17—C18—C19	−1.6 (7)
C7—O1—C8—C9	10.2 (7)	C17—C18—C19—C20	3.9 (7)
C7—O1—C8—C13	−171.0 (4)	C17—C18—C19—I1	−175.0 (4)
O1—C8—C9—C10	−178.9 (4)	C18—C19—C20—C21	−1.8 (7)
C13—C8—C9—C10	2.3 (8)	I1—C19—C20—C21	177.0 (4)
C8—C9—C10—C11	1.7 (7)	C19—C20—C21—C16	−2.5 (8)
C9—C10—C11—C12	−2.5 (7)	C19—C20—C21—C14	176.7 (5)
C9—C10—C11—N1	175.0 (4)	C17—C16—C21—C20	4.7 (8)
C14—N1—C11—C10	154.4 (5)	N3—C16—C21—C20	−175.1 (5)
C14—N1—C11—C12	−28.3 (8)	C17—C16—C21—C14	−174.5 (5)
C10—C11—C12—C13	−0.7 (7)	N3—C16—C21—C14	5.7 (8)
N1—C11—C12—C13	−178.0 (5)	N2—C14—C21—C20	174.4 (5)
C11—C12—C13—C8	4.7 (8)	N1—C14—C21—C20	−7.4 (8)
C11—C12—C13—Cl1	−177.7 (4)	N2—C14—C21—C16	−6.4 (7)
O1—C8—C13—C12	175.6 (5)	N1—C14—C21—C16	171.8 (5)
C9—C8—C13—C12	−5.5 (8)		

### Hydrogen-bond geometry (Å, °)

**Table:**

*D*—H···*A*	*D*—H	H···*A*	*D*···*A*	*D*—H···*A*
N1—H21···N3^i^	0.81 (6)	2.39 (6)	3.128 (6)	151 (6)

Symmetry codes: (i) *x*−1/2, −*y*+1, *z*.
